# Self-replicating segregation patterns in horizontally vibrated binary mixture of granules

**DOI:** 10.1038/s41598-024-55876-y

**Published:** 2024-03-04

**Authors:** Hiroyuki Ebata, Shio Inagaki

**Affiliations:** Department of Physics, Kyushu University, Fukuoka, 819-0395 Japan

**Keywords:** Condensed-matter physics, Nonlinear phenomena

## Abstract

Fluidized granular mixtures of various particle sizes exhibit intriguing patterns as different species segregate and condense. However, understanding the segregation dynamics is hindered by the inability to directly observe the time evolution of the internal structure. We discover self-replicating bands within a quasi-2D container subjected to horizontal agitation, resulting in steady surface waves. Through direct observation of surface flow and evolving internal structures, we reveal the crucial role of coupling among segregation, surface flow, and hysteresis in granular fluidity. We develop Bonhoeffer-van der Pol type equations grounded in experimental observations, reproducing complex band dynamics, such as replication, oscillation, and breathing. It suggests the similarity between pattern formation in granular segregation and that in reaction–diffusion systems.

## Introduction

Granular materials, composed of solid macroscopic particles, play a fundamental role in diverse natural phenomena and industrial processes. Within the industrial sector, significant research efforts have been dedicated to optimizing the efficient handling^1,2^. Granular materials also serve as intriguing examples of systems far from equilibrium due to their dissipative interactions between components. When subjected to mechanical agitation, such as flow, vibration, and rotation, the balance between energy supply and dissipation, gives rise to a rich variety of dissipative structures^3–5^.

Segregation, arising from differences in particle size or density, presents itself as one of the most counterintuitive phenomena^6–9^. When a binary granular mixture is poured between two vertical plates, a remarkable phenomenon known as stratification occurs, wherein alternating layers spontaneously emerge in the quasi-2D sandpile^10^. In a rotating drum containing a binary mixture of grains, the rotation gives rise to alternating bands that progressively merge over time^11,12^. When heavy particles are placed on top of a layer of light particles on a slope, parallel strips aligned with the chute flow direction emerge^13^.

While these segregations are influenced by surface avalanches, it remains unclear how granular rheology and hydrodynamic instability exert a significant influence on shaping the observed patterns. In the context of axial segregation within a rotating drum, the difference in dynamic angles of repose, which correlates with granular fluidity (indicating how easily the granules flow under external forces), is traditionally considered a key parameter. However, certain studies have suggested that axial bands form as a result of bulging in the radial core of small particles, followed by the splitting of the external layer and the subsequent formation of axial bands^14,15^. Due to the limitations of non-invasive observations, it is challenging to verify these hypotheses.

## Results

**Figure 1:**
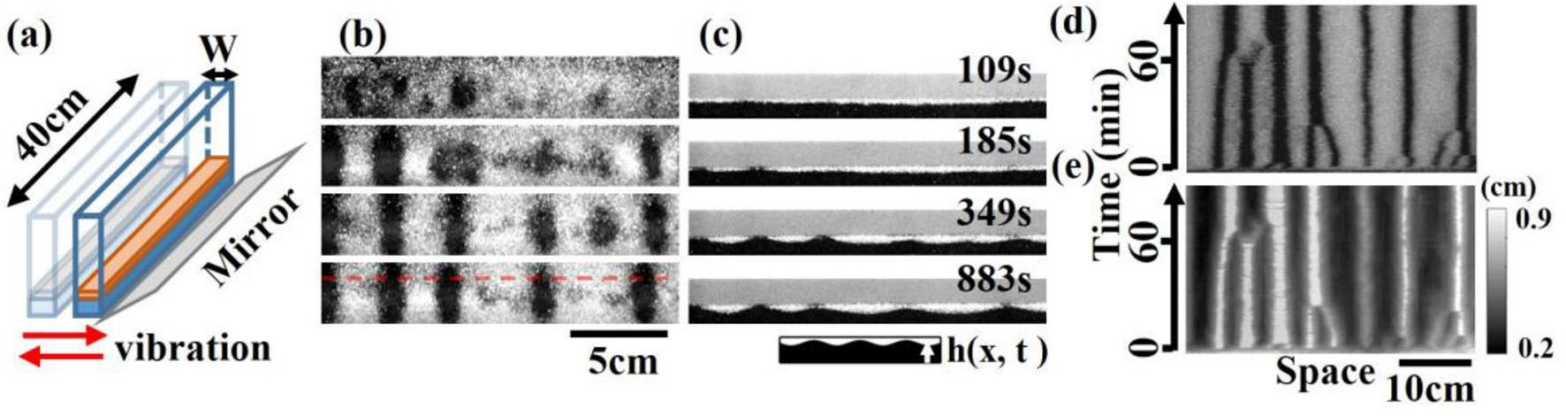
(**a**) Schematic illustration of the experiment. (**b**, **c**) Time series of top (**b**) and side (**c**) views of the band formation. As an initial condition, two granular species are uniformly mixed. The images capture the central 18 cm of the 40 cm container length. (**d**) Spatiotemporal plot created by vertically stacking one-pixel-high horizontal lines extracted from top-view images (see the red-dashed line in (**b**)). (**e**) Height $h\left(x,t\right)$ of the interface between bead and frit layers. The color indicates the height (see colorbar). (**b**–**e**) $W$ = 3.5 cm. Diameter of glass frits and beads are ${D}_{f}$ = 381 μm and ${D}_{b}$ = 868 μm, respectively. Vibration frequency $f$ = 2.8 Hz.

In this study, we conducted experiments in a horizontally shaken narrow channel using spherical and angular particles, exploiting the different fluidity by particle shape (Fig. 1a, see Methods). The vibration was applied parallel to the short axis of the container, with an amplitude of 5 cm (peak-to-peak). The vibration frequency, denoted as $f$, was varied from 0 to 3.0 Hz. Under horizontal vibration with a frequency ($f$ = 2.8 Hz), the granular bed exhibited fluidization, resulting in the formation of a surface wave propagating parallel to the short axis of the container. When two granular species, large glass beads and small glass frits, were initially mixed, this surface wave induced simultaneous segregation in both the vertical and horizontal directions (Fig. 1b,c). Due to the vertical segregation, the granular bed was covered by a layer of large glass beads (white). When the sizes of glass beads (${D}_{b}$) and frits (${D}_{f}$) were 868 and 381 μm, respectively, the bands of glass frits (black) subsequently appeared and merged, leading to the formation of wider bands, as shown in the spatiotemporal plot (Fig. 1d). The observed temporal evolution closely resembled the segregation phenomena typically observed in a rotating drum^11^. From the side view of the container (Fig. 1c), the band formation appeared as an interface instability reminiscent of the Rayleigh–Taylor Instability^13,16^. The interface refers to the boundary between the layer of glass beads and the layer of glass frits. As the undulation of the interface grew, the interface reached the surface, resulting in a splitting behavior of the surface layer of glass beads resembling the formation of “droplets”. The time evolution of the height of the interface between glass beads and frits, $h(x,t)$, exhibited a strong correspondence with that of the surface pattern as shown in Fig. 1d,e.

**Figure 2:**
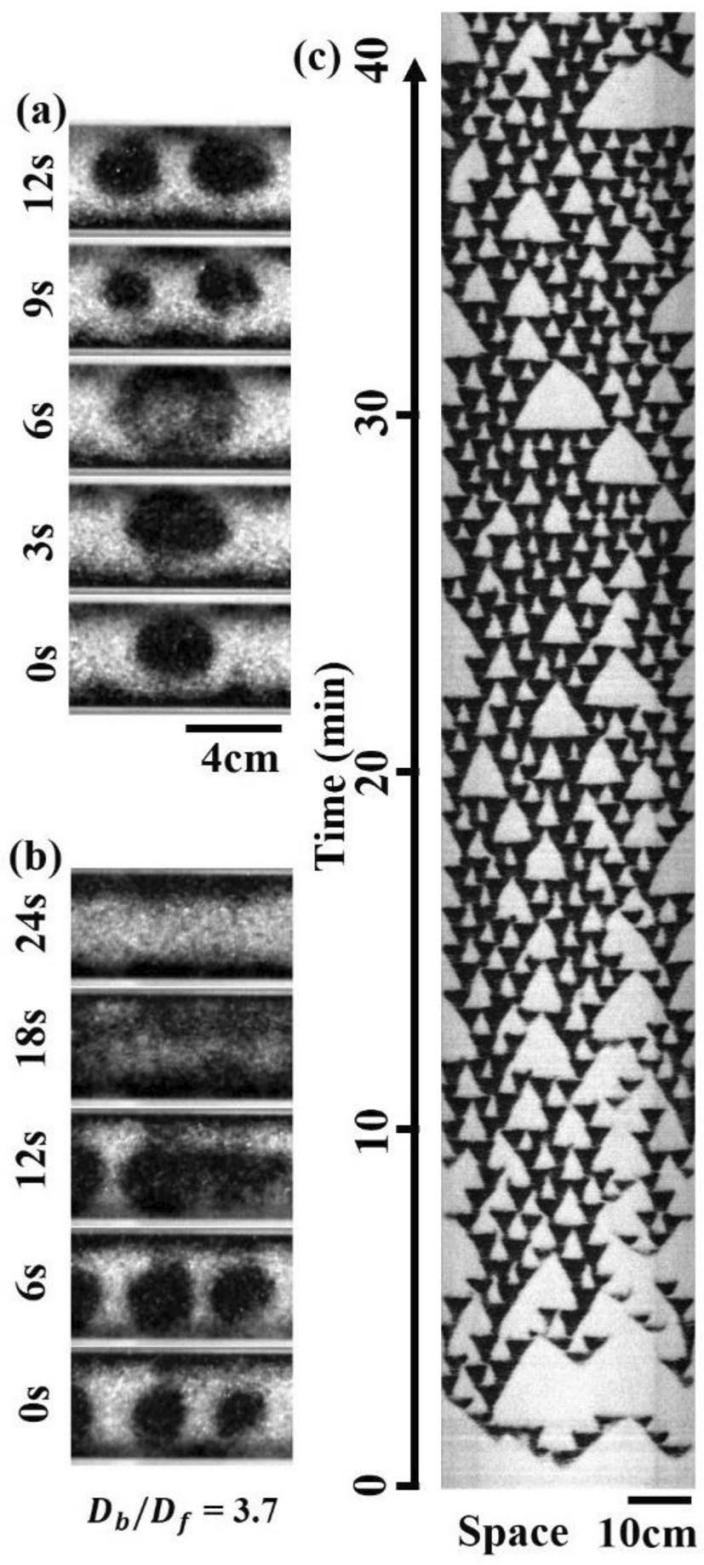
(**a**) Time series of the replicating bands. (**b**) Time series of the pair annihilation. (**c**) Spatiotemporal plots of the replicating bands. (**a**, **b**) ${D}_{f}$ = 337 μm. ${D}_{b}$ =1240 μm. (**c**) ${D}_{f}$ = 254 μm. ${D}_{b}$ = 1090 μm. (**a**–**c**) $f$ = 3.0 Hz. W = 4.0 cm.

In addition to the coarsening of the bands, we observed intriguing self-replication dynamics when ${D}_{b}\sim$ 1100 μm and ${D}_{f}\sim$ 300 μm. Initially, a small band formed and gradually expanded in size. Then, it underwent a splitting process into two distinct bands (Fig. 2a). We also observed that collision between these bands led to their annihilation (Fig. 2b). The replicating dynamics displayed remarkable similarities to replicating patterns observed in reaction–diffusion systems^17,18^ and vertically vibrated suspensions^19^. Due to the successive replication and annihilation of the bands, the spatiotemporal plot in Fig. 2c demonstrated a distinct pattern resembling a Sierpinski gasket observed in the replicating patterns of 1-dimensional reaction–diffusion systems^20,21^. The spatiotemporal plot of the replicating bands exhibits reduced spatial symmetry, which can be attributed to partial replication^22^, partial annihilation^19,23^, and spontaneous creation and annihilation processes occurring within the bands.

**Figure 3:**
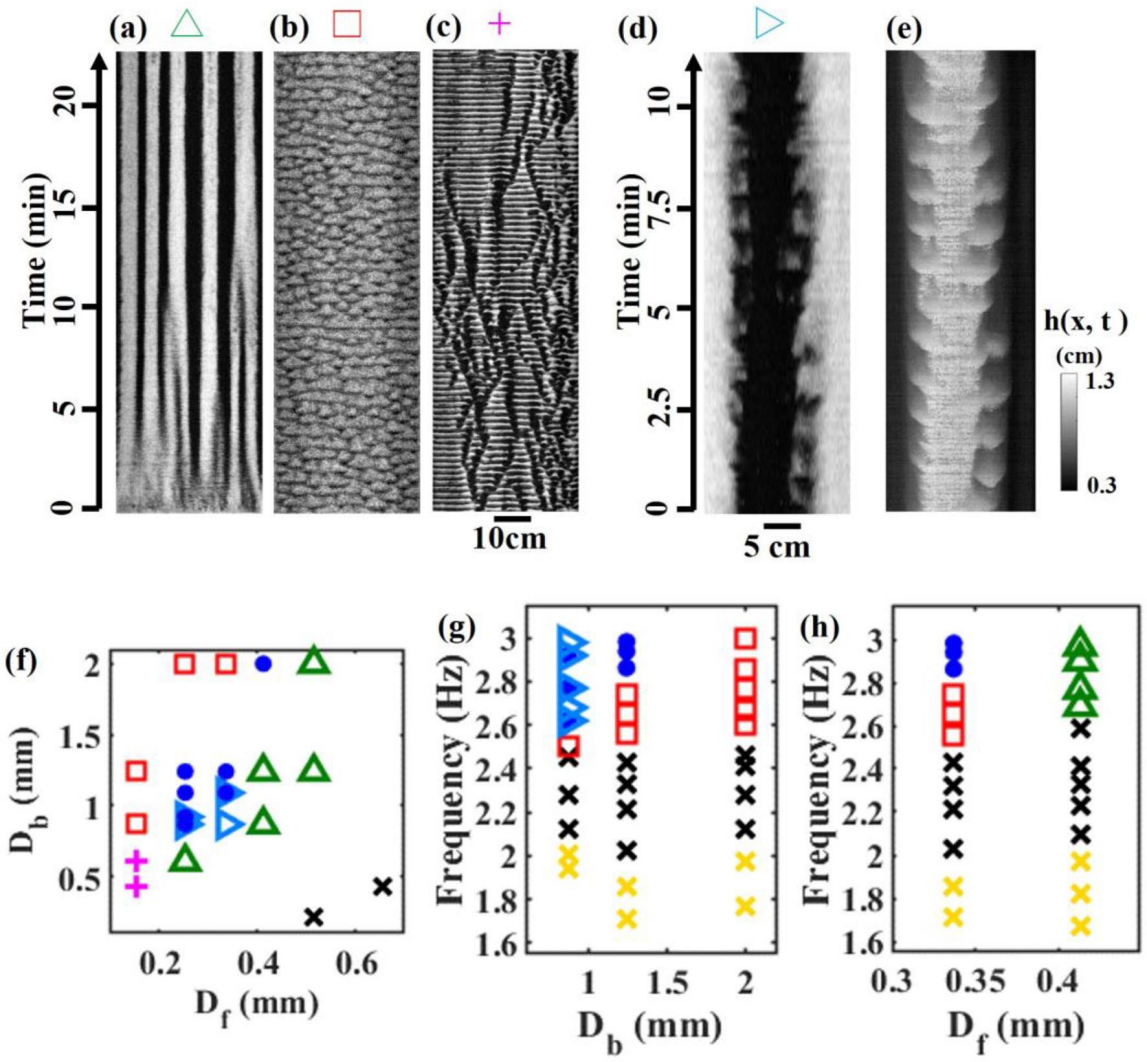
(**a**–**d**) Spatiotemporal plots of the band dynamics. (**e**) Spatiotemporal plot of height $h\left(x,t\right)$ of the interface between bead and frit layers for a breathing band. White band corresponds to the black band in (**d**). The color indicates the height (see colorbar). (**d**, **e**) For clarity, we crop one breathing band. (**f**) Phase diagram of particle size combination (${D}_{f}$ and ${D}_{b}$) demonstrating segregated patterns. (**g**, **h**) Dependence of band pattern on vibration frequency with different ${D}_{b}$ (**g**) and ${D}_{f}$ (**h**). (**a**–**h**) Blue circle: replication. Red square: oscillation. Green triangle: coarsening. Cyan triangle: breathing. Pink cross: traveling wave. Black cross: no bands. Yellow cross: no flow. (**a**) ${D}_{f}$ = 413 μm. ${D}_{b}$ = 868 μm. (**b**) ${D}_{f}$ = 337 μm. ${D}_{b}$ = 2000 μm. (**c**) ${D}_{f}$ = 153 μm. ${D}_{b}$ = 605 μm. (**d**, **e**) ${D}_{f}$ = 337 μm. ${D}_{b}$ = 868 μm. (**g**) ${D}_{f}$ = 337 μm. (**h**) ${D}_{b}$ = 1240 μm. (**a**–**f**) $f$ = 3.0 Hz (**a**–**h**) W = 4.0 cm.

Figures 3a–e depict further band dynamics arising from variations in particles sizes (see supplemental movies). Figure 3f presents a phase diagram illustrating the range of band patterns that arise depending on the combination of grain sizes. In the region of green triangle in Fig. 3f, the band dynamics underwent a coarsening process as observed in Fig. 1d. Eventually, several bands persisted, reaching a steady state. As we increased ${D}_{b}$ or decreased ${D}_{f}$, the coarsening state bifurcated into oscillatory patterns through replicating bands. In the marginal parameter between coarsening state and replicating bands, we also found breathing bands, where the width of the band oscillated (cyan triangle in Fig. 3d–g). In the region where the bule circles and cyan triangles coexist in Fig. 3f,g, replicating and breathing bands appeared simultaneously. When the glass beads and frits were both small, the system became disrupted. The traveling waves of glass beads and frits were created along one long side of the container and propagated to the other side, resulting in the oscillatory pattern in the spatiotemporal plot (pink cross in Fig. 3c,f). Band formation did not occur when ${D}_{b}/{D}_{f}$ < 1, indicating that band formation requires the spherical beads to be larger than the angular frits in size.

Figure 3g shows the dependence of the band pattern on vibration frequency and ${D}_{b}$ with fixed ${D}_{f}$ = 337 μm. At low vibration frequencies, the granular bed remained stationary, and no surface flow was induced. With an increase in vibration frequency, the flow of larger particles initiated, but band formation did not yet occur (black cross). However, above a certain critical frequency, narrow bands started to appear and disappear in an oscillatory manner. The onset frequency of oscillatory bands marginally depended on ${D}_{b}$. With further increase in vibration frequency, the bands began to breath or replicate, leading to a more complex pattern for ${D}_{b}$ $\le$ 1240 μm. When ${D}_{b}$ was large, only oscillatory bands appeared. In the case of larger ${D}_{f}$ with ${D}_{b}$ = 1240 μm, coarsening process emerged above a critical frequency (Fig. 3h). These observations emphasize the significance of surface flow dynamics, which serve as a reflection of the rheology of shaken dry granules^24,25^.

**Figure 4:**
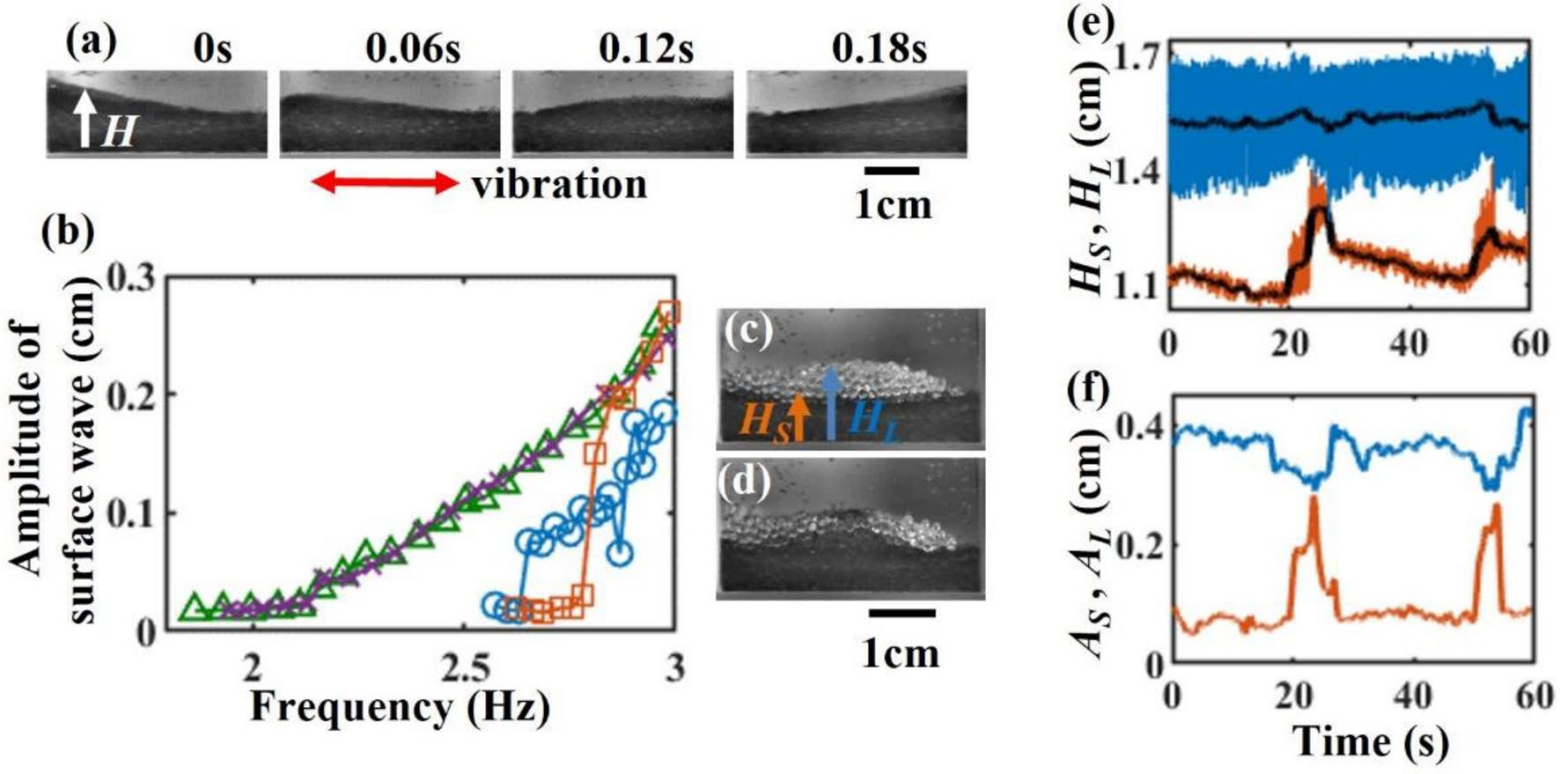
(**a**) Time series of the surface traveling wave of a glass frits' bed during a vibration cycle. The images were captured laterally from the short side of the container. (**b**) Amplitude of surface wave vs. vibration frequency: Green triangle (decreasing $f$, beads' bed), Purple cross (increasing $f$, beads' bed), Blue circle (decreasing $f$, frits' bed), Red square (increasing $f$, frits' bed). (**c**, **d**) Typical lateral view of the segregated granular mixture without band (**c**) and with band (**d**). (**e**) Time evolution of ${H}_{L}$ (blue) and ${H}_{S}$ (orange). The replicating band appeared occasionally. (**f**) Time evolution of the oscillation amplitude ${A}_{L}$ (blue) and ${A}_{S}$ (orange) of ${H}_{L}$ and ${H}_{S}$. (**a**–**f**) ${D}_{f}$ = 337 μm. (**b**–**f**) ${D}_{b}$ = 1240 μm. (**c**–**f**) $f$ = 2.9 Hz. (**a**–**f**) $W$ = 4.0 cm.

To characterize the surface flow of the granular bed, we measured the amplitude of the surface wave of the granular bed using a single species (Fig. 4a). We captured the images of the granular bed laterally from the short side of the container and then determined the height of the surface of the entire granular bed. The wave amplitudes were defined as the standard deviations of the height over one complete vibration cycle. Figure 4b presents the frequency ramp test, where we initially applied a high vibration frequency and gradually reduced it until the surface flow ceased. We then incrementally increased the frequency back to its original value. For the glass beads, we observed the onset of the bead layer flow at a relatively low vibration frequency. The surface wave amplitude did not show a pronounced hysteresis with frequency, as the glass beads readily rolled on the surface of the granular bed. In contrast, the layer of glass frits required a higher vibration frequency to initiate flow, due to the angular shape of the particles. We observed a strong hysteresis in the surface wave amplitude (For different ${D}_{b}$ and ${D}_{f}$, see Fig. S2 in supplementary information). Even at sufficiently large vibration frequencies, the surface waves displayed branches with higher and lower amplitudes, indicating the presence of hysteresis in the flow which is commonly observed in frictional grains^25–27^. Figures 3g and 4b clearly illustrate that the onset frequency ${f}_{band}$ of the band formation is close to the lowest frequency ${f}_{flow}$ at which flow occurs in the glass frits layer ($f$ ~ 2.5—2.6 Hz). Note that ${f}_{band}$ is 0.05–0.1 Hz smaller than ${f}_{flow}$ (Figs. 3g,h, 4b, and Fig. S2 in supplementary information). In Fig. 3, we initially located all glass beads on the frits’ bed. Thus, to initiate the band formation, the frits must flow and move into the surface glass beads’ layer. The relation ${f}_{band}\le {f}_{flow}$ suggest that in Fig. 3, the flow of the glass beads activates the movement of the frits.

To reveal the interaction between the surface flow and band dynamics, we measured the surface wave dynamics during the formation of bands in the vertically segregated granular mixture of beads and frits. We focused on the surface height of the bead layer ${H}_{L}$ and the interface height between the bead and frit layers ${H}_{S}$ (Fig. 4c,d). Figure 4e demonstrates the time series of ${H}_{L}$ (blue line), ${H}_{S}$ (orange line), and their time-averaged values (black lines) for replicating bands. The oscillation of ${H}_{L}$ represents the propagation of surface waves from one side to the other (Fig. 4a). Since the surface was predominantly covered by glass beads, ${H}_{L}$ consistently remained larger than ${H}_{S}$. As the band formed, ${H}_{S}$ sharply increased and approached ${H}_{L}$. In addition, the presence of the bands significantly influenced the amplitude of surface flow, ${A}_{L}$ and ${A}_{S}$, which represent the standard deviations of ${H}_{L}$ and ${H}_{S}$ over one vibration cycle (Fig. 4f). When a band appeared near the wall, we observed a slight decrease in ${A}_{L}$ and a pronounced increase in ${A}_{S}$, resembling a pulse-like behavior seen in excitable media^28^. This substantial discrepancy between ${A}_{L}$ and ${A}_{S}$ suggests the presence of shear stress at the interface. Thus, we anticipated that the flow of the glass frits would be significantly agitated by the shear flow exerted by the layer of glass beads.

**Figure 5:**
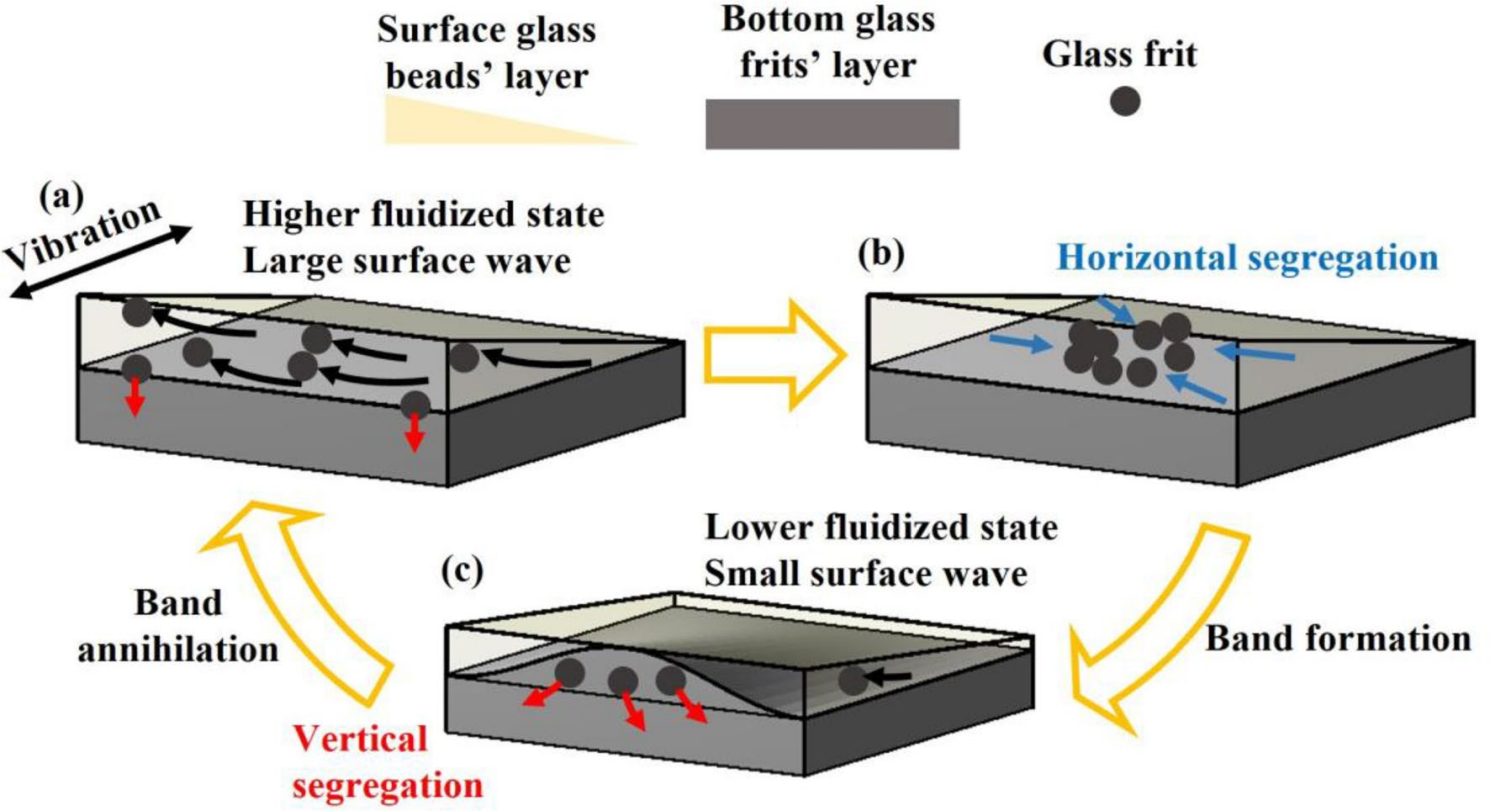
Schematic illustration of suggested mechanism of band formation and annihilation. Light yellow area represents surface glass beads’ layer. Dark gray area represents bottom glass frits’ layer. (**a**) Since the number of frits is small in surface layer, large surface wave is induced due to vibration. Large wave blows frits up from bottom layer (black arrows). (**b**) The frits in surface layer accumulate due to horizontal segregation (blue arrows). Then, a band forms. (**c**) Increasing number of frits in the surface layer causes decrease of fluidity and amplitude of the surface wave. As a result, the particle supply from the glass frits' layer almost stops. Finally, band disappears due to vertical segregation (red arrows).

The experimental results suggest an intricate interplay between the surface wave and band dynamics (Fig. 5). The shear flow at the interface gradually entrains and incorporates the glass frits into the highly fluidized layer of beads (Fig. 5a). The increasing number of frits in the bead layer leads to the growth of the band owing to segregation in the horizontal direction (Fig. 5b)^29,30^. However, as indicated in Fig. 4b, the highly fluidized state of the glass frits is metastable^25^. The condensation of the frits decreased the amplitude of the total surface wave owing to the low fluidity and triggered the jump into the lower fluidized state, which decreased ${A}_{S}$ and ${A}_{L}$. Consequently, the particle supply from the glass frits' layer decreased or stopped (Fig. 5c). Then, the glass frits on the surface were absorbed into the underlying basal frits layer due to vertical segregation^31^, resulting in the annihilation of frit bands.

To develop a phenomenological model to describe the band dynamics, we introduce two key variables: the phase $\phi$ of the surface bead layer and the state of flow $\psi$ of the surface layer (the region between *H*_*L*_ and *H*_*S*_ in Fig. 4c). The remaining region below *H*_*S*_ in Fig. 4c is considered as the particle bath. We propose that the phase $\phi$ is positively correlated with the number density of frits in the surface bead layer; $\phi$ < 0 and $\phi$ > 0 represent bead-rich and frits-rich phases, respectively. As Fig. 4c,d show, the particles could reciprocate between surface layer and particle bath. Thus, $\phi$ in our model does not conserve. We also assume that the state of flow $\psi$ is directly linked to the magnitude of the surface flow, with an increase in $\psi$ corresponding to an increase in the amplitude of the surface wave. Thus, $\psi \ll$ 0 indicates that the surface flow stops. The dynamics of $\phi$ is assumed to be linearly dependent on $\psi$, as the small particles were entrained into the surface fluidized layer caused by the surface flow. When the flow $\psi$ is constant, $\phi$ is found to relax to a certain value that is a function of $\psi$. Thus, the model equation is written as

1 $$\begin{array}{c}{\tau }_{\phi }\frac{d\phi }{dt} =\psi -\gamma \phi + I + {D}_{\phi }{\nabla }^{2}\phi ,\end{array}$$

where ${\tau }_{\phi }$, $\gamma$, $I$, and ${D}_{\phi }$ are the typical time scale of evolution of $\phi$, the relaxation rate, offset (leak) of the phase from particle bath, and diffusion coefficient, respectively. Here, we introduce diffusion term because the surface flow induces diffusion of the granular materials perpendicular to the flow direction^32–34^.

**Figure 6:**
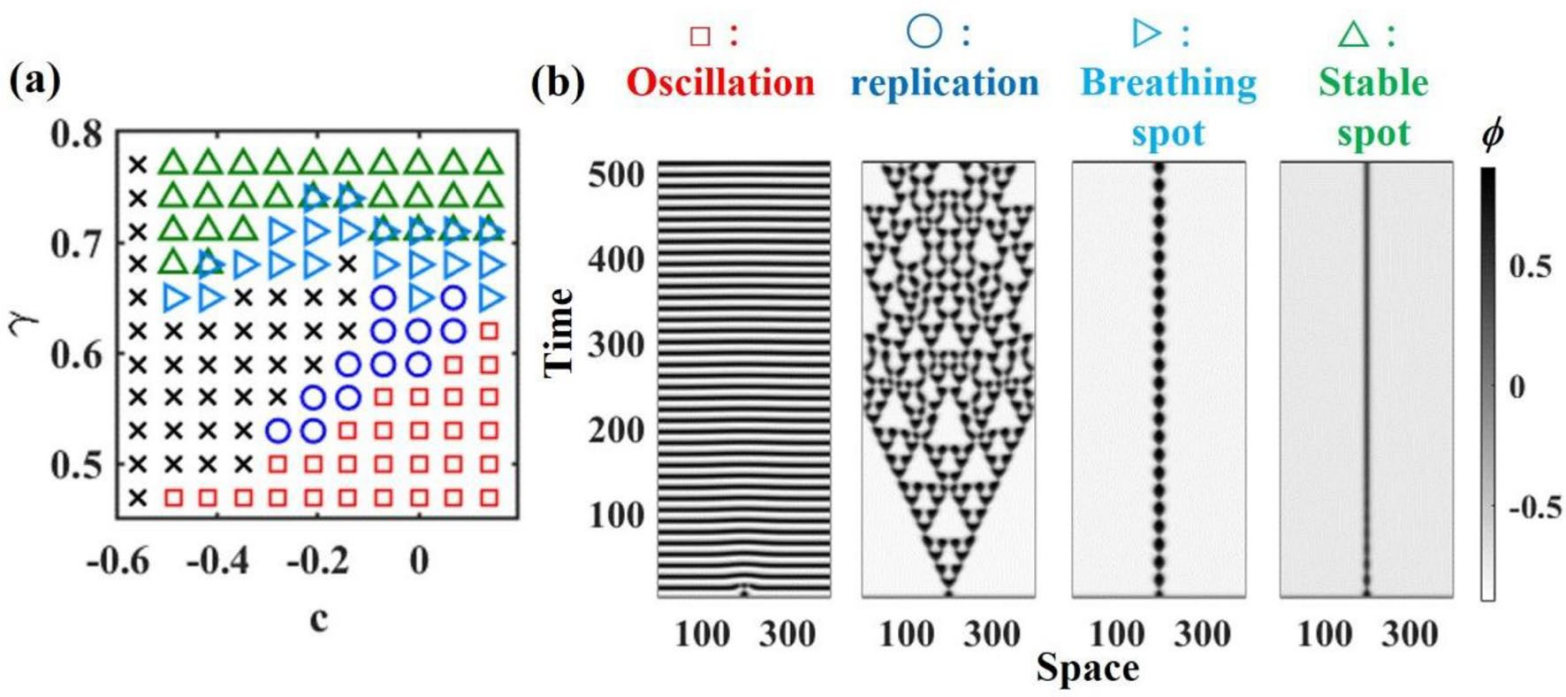
(**a**) Phase diagram of patterns obtained from Eqs. (1) and (2). Red square: oscillating pattern. Green triangle: stable spot. Blue circle: replicating spot. Black cross: uniform state. ${\tau }_{\psi }$ = 0.49, $d$ = 2.25, ${\tau }_{\phi }$ = 2.25, $I$ = 0.1, $a$ = 5, ${D}_{\phi }$ = 22.5, ${D}_{\psi }$ = 1. (**b**) Typical spatiotemporal plot of the oscillating pattern, replicating spots, a breathing spot, and a stable spot. The color bar represents the $\phi$ value, with darker colors indicating larger $\phi$ values.

We consider that the flow $\psi$ depends on the phase $\phi$ of the surface fluidized layer because an increase in frits in the fluidized layer weakens the surface wave (Fig. 4f, blue line). Furthermore, $\psi$ can be bistable owing to the strong hysteresis of the frits flow^25,35^ (Fig. 4b and f). For simplicity, we introduce a cubic nonlinear term for the time-evolution equation of $\psi$ to implement bistability as

2 $$\begin{array}{c}{\tau }_{\psi }\frac{d\psi }{dt} = a\psi \left(1+\psi \right)\left(1-\psi \right) +c - d\phi + {D}_{\psi }{\nabla }^{2}\psi ,\end{array}$$

where ${\tau }_{\psi }$, $c$ and ${D}_{\psi }$ are the typical time scale of evolution of $\psi$, the offset and diffusion coefficients, respectively. $a$ is a positive constant, with larger values of $a$ enhancing the stability of the bistable points in the flow state. $c$ regulates the bi-stability of the flow; an increase in $c$ results in higher amplitude waves becoming more stable. $d$ represents the extent to which the frits, $\phi$, attenuate the surface flow, $\psi$. Equations (1) and (2) represent Bonhoeffer-van der Pol type reaction–diffusion equations, which have been known to exhibit self-replicating pulses^21^. To align with the observed replicating bands in the experiment, we chose parameter values ensuring a close match in both size and replication period. This consideration takes into account the simulation's operation on time and spatial scales of seconds and millimeters, respectively (see Figs. 2 and 6).

Figure 6a depicts the phase diagram of the model, revealing the bifurcation of the pattern from oscillating pattern to a stable spot formation (Fig. 6b) as the relaxation rate $\gamma$ increases. By varying c, we investigate how changes in the bi-stability of the flow state impact pattern formation. For our specific parameter set, replication occurs only under specific combinations of $\gamma$ and $c$. Comparing the experimental results (Fig. 3) with the simulation (Fig. 6), our model reproduces bifurcations among oscillatory, replicating, and breathing patterns. Note that our one-dimensional model cannot describe the travelling wave in the experiment (pink cross in Fig. 3), because the dynamics of travelling wave was two-dimensional (see supplementary movie).

## Discussion

In our phenomenological model, we successfully reproduce replicating and oscillatory band dynamics. However, one remaining challenge is the emergence of stable spots in the simulation, whereas the experimental observations revealed a coarsening process. Our model currently lacks the ability to replicate this coarsening process. Prior research in reaction–diffusion systems has demonstrated that the introduction of mass conservation can lead to coarsening processes^36^, while a mild violation of mass conservation can interrupt the coarsening, leaving stable spots or domains after the process^37^. In our one-dimensional model, we specifically focus on the time evolution of particles in the surface region (${H}_{S}$ to ${H}_{L}$ in Fig. 4e). Since particles can reciprocate between the surface and bottom regions (0 to ${H}_{S}$ in Fig. 4e), mass conservation is not upheld in general. However, under conditions of strong segregation in the depth direction, where nearly all glass beads remain in the surface region, mass conservation virtually holds. In such instances, the thickness evolution of the glass bead layer could be modeled using mass-conserving equations, such as those found in thin liquid film equations or the Chan-Hillard model^38^, both of which exhibit coarsening processes. Therefore, a potential improvement to our model is the incorporation of mass conservation, with its violation being contingent on the strength of the segregation.

Another important consideration is the relationship between physical property of the granules and the parameters of the model. In rotating drums with spherical particles, the particle size differences crucially regulate the onset and strength of the segregation^39^. For example, the particle size ratio larger than 6 has been shown to suppress radial segregation and the formation of bands^39,40^. In our model, $\gamma$ characterizes the rate at which segregation progresses, suggesting a potential negative correlation with particle size differences when these differences are substantial. Moreover, examining the dependence of the frits' surface wave on vibration frequency revealed increased stability of the fluidized state with higher frequencies (Fig. 4b and S2 in supplementary information). Consequently, the parameter c, which governs the bi-stability of the flow, may have a positive relation to vibration frequency, as larger c stabilizes the flow state. However, direct comparison between experimental results (Fig. 3) and simulations (Fig. 6) remains challenging due to potential dependencies of other model parameters on particle size differences and vibration frequency. To establish a direct connection between the model parameters and physical properties, it is imperative to develop a model derived from individual particle dynamics and granular rheology.

Previous studies have reported the presence of coarsening bands in horizontally vibrated monolayer granular mixtures, where the vibration amplitude was significantly smaller than the container size^30,41,42^. Theoretical studies and simulations using the distinct element method predicted that the inertial effect and frictional force between the particle and container surface play a crucial role in band formation in monolayer granular mixtures^43,44^. In our study, the phenomenological model suggests that the complex dynamics of pattern formation originate from the bistability of the granular flow. Thus, the bulk rheology arising from particle–particle interactions serves as a fundamental mechanism underlying the observed band dynamics.

Drawing inspiration from the segregation phenomena in a rotating drum, we conducted experiments to investigate the dynamics of band formation in a granular system comprising two distinct types of grains with different fluidity. As we show in Figs. 3g and 4b, the surface flow regulated the dynamics of the band. In a rotating drum with a high fill level, the cross-sectional images of the granular mixture suggested that the radial and axial segregation were driven by the shear induced by the surface flow^45,46^. Previous studies have proposed that axial segregation in a rotating drum can be considered as interfacial instability between the layer of large particles and that of small particles^15,47^. Our observations from the side-view (Fig. 1c) also implied that the band formation in our system was originated from the interfacial instability.

In the case of half-filled rotating drums, side walls of the container can induce the segregation and flow, which is called as end wall effect^48,49^. When the length of the drum is short, end wall effect dominantly regulates the segregation dynamics, and the bands appear near the wall^50^. In our experiment, coarsening bands made from glass frits consistently appeared at the ends of the long axis of the container within 10 min. This behavior resembles that of coarsening bands in rotating drums, indicating that the end wall influences the coarsening process. However, no such effect was observed in the replicating bands and oscillatory pattern for up to 1–3 h. This might be attributed to the metastable nature of replicating and oscillatory bands, which cannot persist even near the end wall (Fig. 4e). The strength of the end wall effect may depend on particle size differences, as indicated by the phase diagram (Fig. 3f). Additionally, the impact of the walls on the longer sides remains unclear. To address these uncertainties, further experiments involving long-term measurements and containers of different widths and lengths are needed.

It is also known that a mixture of spherical glass beads with different particle sizes formed clear bands in rotating drums^39^, whereas we only observed subtle bands with the same combination of glass beads in our system (see supplementary information). The effect of particle shape on fluidity necessitates further investigation as an important parameter. Recently, it was reported that the size and density differences between two types of particles dominantly determine the onset of axial segregation in a rotating drum^39^. In our experiments, when granules with different densities were used, we observed additional band dynamics in the form of zig-zag droplets (see supplementary information). Our preliminary findings indicate that the pattern dynamics of mixtures of spherical beads also bifurcate depending on the frequency, particle size difference, and density difference (Fig. S3 in the supplementary information). However, the specific contributions of granular fluidity^25^, density difference^13^, and size differences to the segregation dynamics are not yet fully understood. In particular, the bifurcation of band patterns in mixtures of spherical beads requires further investigation in future studies. Our system enables us to explore the detailed time evolution of the interfaces between granular beds. Further investigations will enhance our understanding of the mechanisms driving segregation patterns induced by surface flows.

## Methods

We used a reciprocal shaker NR-10 (TAITEC, Japan). The initial depth of the granular bed was approximately 1 cm, while the container's dimensions were 40 cm in length, as depicted in Fig. 1a. In Fig. 1b–e, the width of the container, $W$, was set to 3.5 cm, while for the remaining figures, $W$ was set to 4.0 cm. To facilitate observation of the internal structures, we placed a mirror at the side of the container, enabling simultaneous monitoring of surface and internal dynamics. The imaging was performed using a CCD camera and a high-speed camera (HAS U-1, Detect). In the case of the CCD camera, images were acquired at a specific vibration phase by tracking the container's position using a laser sensor. The observation time of the experiment was 25–180 min. To prevent slipping of the granular bed, glass beads with a diameter of 0.8 mm were glued to the bottom of the container. To eliminate the influence of density difference, we utilized spherical glass beads (white color, density of 2.56 g/cm^3^) with the average size, ${D}_{b}$, ranging from 400 to 2000 μm, and angular glass frits (black color, density of 2.57 g/cm^3^) with the average sizes, ${D}_{f}$, ranging from 150 to 650 μm (refer to supplementary information for further details). The size distribution of the sieved glass frits was measured by Seishin Enterprise Co., Ltd. using laser diffraction and scattering (LMS-3000, Malvern Panalytical). The densities of the glass beads and frits were measured by Seishin Enterprise Co. Ltd. using the picnometer method. To ensure high reproducibility of the experiments, we arranged a layer of large glass frits on top of a layer of small glass beads, unless otherwise specified. For the experiment involving the initially mixed state (Fig. 1), we thoroughly blended 132 g of glass frits with an equal amount of glass beads. Subsequently, we evenly distributed the granular mixture by sprinkling it into a 3.5 cm × 40 cm rectangular container. For the experiment with the initially segregated state (Figs. 2, 3 and 4), we first placed a layer of glass frits (151.5 g) in a 4 cm × 40 cm rectangular container and then sprinkled 61.0 g of glass beads on the frit layer.

### Supplementary Information

Supplementary Information.

## Data Availability

All data needed to evaluate the conclusions in the paper are present in the paper and/or the Supplementary Materials. Additional data related to this paper may be requested from the corresponding author.
