# Supplementary material for: Self-replicating segregation patterns in horizontally vibrated binary mixture of granules
Source: Sci Rep. 2024 Mar 4;14:5329. doi: 10.1038/s41598-024-55876-y (PMC10912327; doi:10.1038/s41598-024-55876-y)

**Supplementary Information for**

**“Self-replicating segregation patterns**

**in horizontally vibrated binary mixture of granules”**

Hiroyuki Ebata, Shio Inagaki

Graduate school of Science, Kyushu University, 744 Motooka, Nishi-ku, Fukuoka 819-0395, Japan

**A. Movie captions**

**coarsening.avi : Time-lapse movie of coarsening bands.**

Length of the movie corresponds to 147 min. Screen width corresponds to 40 cm. Images were acquired at a specific vibration phase.

**breathing.avi : Time-lapse movie of coarsening bands.**

**oscillation.avi : Time-lapse movie of oscillating bands.**

**replication.avi : Time-lapse movie of replicating bands.**

**travelling wave.avi : Time-lapse movie of travelling wave pattern.**

Length of the movie corresponds to 22 min. Screen width corresponds to 40 cm. Images were acquired at a specific vibration phase.

**rep_one_band.avi : Time-lapse movie of one replicating band.**

Length of the movie corresponds to 37 sec. Screen width corresponds to 8.6 cm. Images were acquired at a specific vibration phase.

**B. Details of the experimental condition**

A typical image of the glass frits is shown in Fig. S1. The average diameter of the sieved glass frits is listed in Table S1.


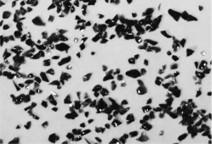


Fig. S1. Image of the glass frits.

| *D_f_* (μm) | 153 | 254 | 337 | 413 | 516 | 655 |
| --- | --- | --- | --- | --- | --- | --- |

Table S1. List of average diameters *D_f_* of sieved glass frits.

**C. Amplitude of surface wave vs. vibration frequency for other particles**

Fig. S2. Amplitude of surface wave vs. vibration frequency (a, b) Glass frits’ bed with $D_{b}$ = 868 μm (a) and $D_{b}$ = 2000 μm (b). Green triangle : decreasing $f$. Purple cross : increasing $f$. (c) Glass frits’ bed with $D_{f}$ = 413 μm. Blue circle :decreasing $f$. Red square : increasing $f$. (a - c) $W$ = 4.0 cm.


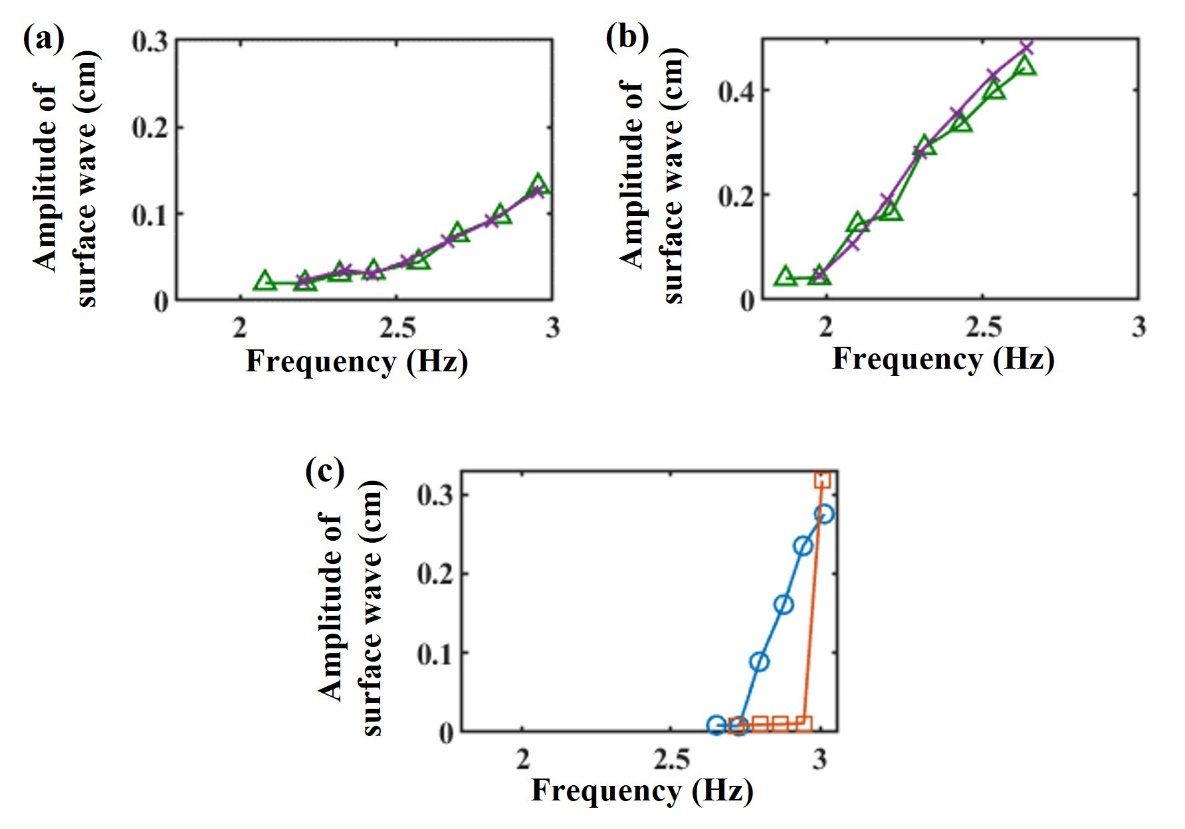


**D. Experiment with spherical particles**

When a combination of spherical glass beads (0.4 mm) and spherical aluminum oxide beads (1.0 mm, 3.6 g/cm^3^) was used, band formation emerged. Specifically, at a moderate vibration frequency (approximately 2.5 Hz), bands of aluminum oxide beads alternately localized around the longer sides of the container, as shown in Fig. S3 (a) (upper image). However, upon increasing the size of aluminum oxide beads to 2.0 mm, clear segregation was not observed (Fig. S3 (a), lower image). In the case of glass beads with different sizes (0.4 mm and 1.1 mm), subtle bands were observed above a certain frequency (Fig. S3 (b)), with no bands appearing at lower frequencies. Enhanced image contrast revealed coarsening dynamics at 2.9 Hz (Fig. S3 (c)). The side view of the bands of aluminum oxide beads (Fig. S3(d)) shows the formation of 'droplets' composed of large particles in the depth direction, similar to the observation in the side view of the bands composed of glass beads (Fig. 1(c) in the main text). On the other hand, we did not observe clear droplet formation for subtle bands in the glass beads of different sizes (Fig. S3 (e)).

Fig. S3. (a) Pattern formation in the combination of glass beads (0.4 mm) and aluminum oxide beads with different particle size (upper image: 1.0 mm. lower image: 2.0mm). Vibration frequency was 2.5 Hz. White region represents aluminum oxide beads. (b) Surface of the combination of glass beads of two different sizes (0.4 and 1.1 mm). The vibration frequencies were 2.5 Hz (upper image) and 2.9 Hz (lower image). While no pattern appears at 2.5 Hz, subtle bands appeared at 2.9Hz. (c) Spatiotemporal plot of Fig. S2 (b). We intensified the image contrast. Darker and brighter regions represent the large and small particle-rich areas, respectively. (d) Side (upper image) and top (lower one) views of one band in Fig. S2 (a). Side view reflects the droplet formation of aluminum oxide beads. (e) Side (upper image) and top (lower one) views of one band in Fig. S2 (b). Red arrow indicates a band.


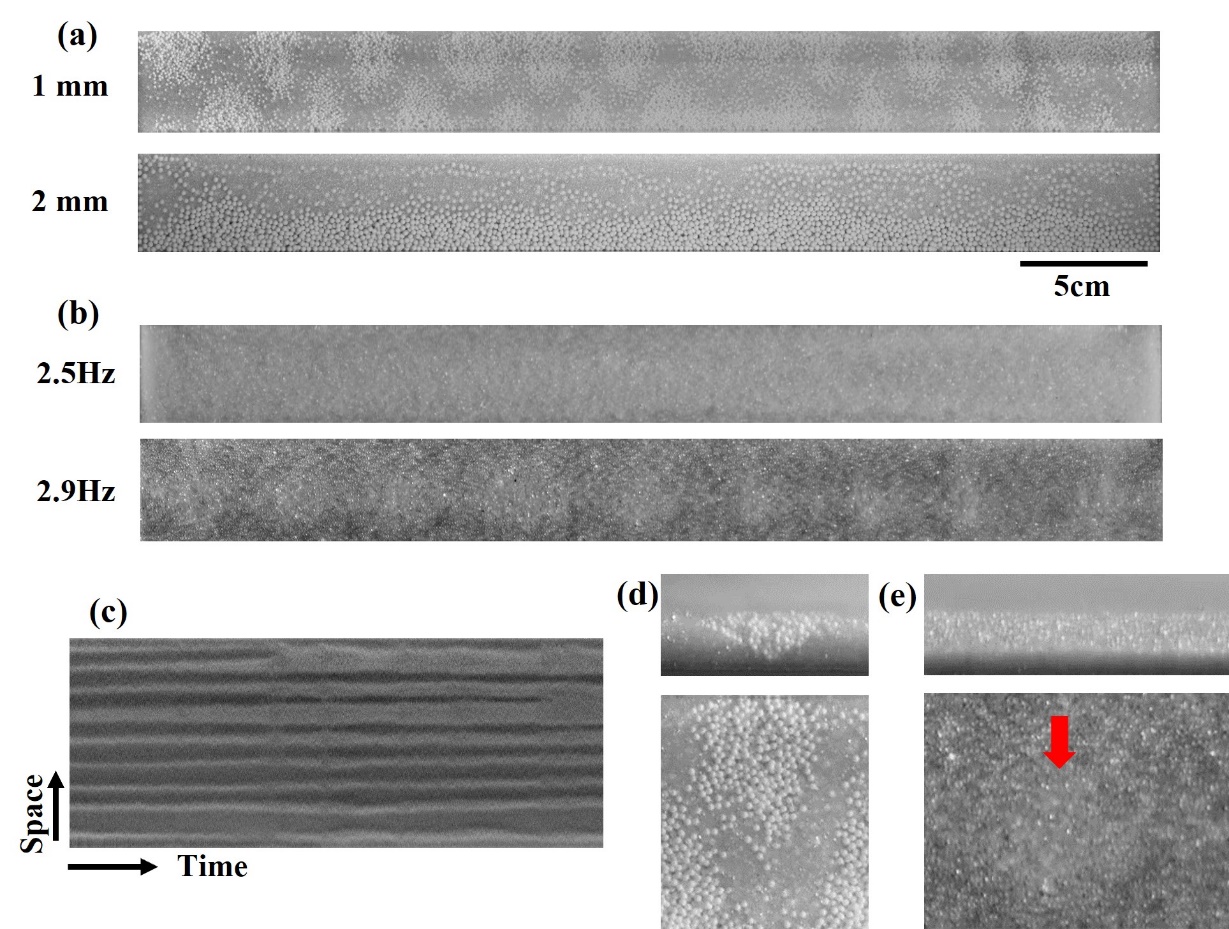

Supplement: Supplementary file 1 — Supplementary Information. [file 41598_2024_55876_MOESM1_ESM.docx]
